# Dr. W. L. Willett

**Published:** 1913-02

**Authors:** 


Dr. W. L. Willett died at his home in Buffalo, January 5. He was formerly Superintendent of the N. Y. State Custodial Asylum at Newark, and was also postmaster of Newark, N. Y., for four years. He served as a private and corporal during the Civil War. He was 72 years old.

